# *In silico* abstraction of zinc finger nuclease cleavage profiles reveals an expanded landscape of off-target sites

**DOI:** 10.1093/nar/gkt716

**Published:** 2013-08-14

**Authors:** Jeffry D. Sander, Cherie L. Ramirez, Samantha J. Linder, Vikram Pattanayak, Noam Shoresh, Manching Ku, Jennifer A. Foden, Deepak Reyon, Bradley E. Bernstein, David R. Liu, J. Keith Joung

**Affiliations:** ^1^Molecular Pathology Unit, Center for Cancer Research, Massachusetts General Hospital, Charlestown, MA 02129, USA, ^2^Center for Computational and Integrative Biology, Massachusetts General Hospital, Charlestown, MA 02129, USA, ^3^Department of Pathology, Harvard Medical School, Boston, MA 02115, USA, ^4^Program in Biological and Biomedical Sciences, Harvard Medical School, Boston, MA 02115, USA, ^5^Department of Chemistry and Chemical Biology, Harvard University, Cambridge, MA 01238, USA, ^6^Broad Institute of MIT and Harvard, Cambridge, MA 02142, USA and ^7^Howard Hughes Medical Institute, Chevy Chase, MD 02815, USA

## Abstract

Gene-editing nucleases enable targeted modification of DNA sequences in living cells, thereby facilitating efficient knockout and precise editing of endogenous loci. Engineered nucleases also have the potential to introduce mutations at off-target sites of action. Such unintended alterations can confound interpretation of experiments and can have implications for development of therapeutic applications. Recently, two improved methods for identifying the off-target effects of zinc finger nucleases (ZFNs) were described–one using an *in vitro* cleavage site selection method and the other exploiting the insertion of integration-defective lentiviruses into nuclease-induced double-stranded DNA breaks. However, application of these two methods to a ZFN pair targeted to the human *CCR5* gene led to identification of largely non-overlapping off-target sites, raising the possibility that additional off-target sites might exist. Here, we show that *in silico* abstraction of ZFN cleavage profiles obtained from *in vitro* cleavage site selections can greatly enhance the ability to identify potential off-target sites in human cells. Our improved method should enable more comprehensive profiling of ZFN specificities.

## INTRODUCTION

Gene-editing nucleases, such as zinc finger nucleases (ZFNs), transcription activator-like effector nucleases (TALENs) and clustered regularly interspaced short palindromic repeats (CRISPR)/CRISPR-associated (Cas) nucleases, can be used to create targeted sequence alterations with high efficiencies in numerous cell types and organisms (1–7). Repair of nuclease-induced double-stranded breaks can be exploited to introduce either insertion/deletion (indel) mutations via non-homologous end-joining (NHEJ) or specific sequence alterations from a donor template via homology-directed repair (1,2). Comprehensive delineation of unintended off-target mutations is important for customized nucleases in many biological applications and will be essential for developing therapeutic strategies based on these proteins.

Two different methods have recently been described for characterizing the genome-wide specificities of ZFNs (8,9), but neither study comprehensively identified the full-spectrum of possible off-target mutations. One method, previously developed by Liu and colleagues, used an *in vitro* cleavage site selection assay to identify sequences from a large partially degenerate library (based on the intended target DNA site) that can be cleaved by ZFNs. *In vitro* selections with a *CCR5*-targeted ZFN pair identified 36 potential off-target cleavage sites that occur in the sequence of the human genome; analysis of these sites in human cells in which CCR5-targeted ZFNs had been expressed revealed nine *bona fide* off-target sites (8). Another approach, described by von Kalle and colleagues, exploited the incorporation of integrase-deficient lentivirus (IDLV) DNAs into nuclease-induced double-stranded breaks to map ZFN cleavage sites in human cells (9). Application of this approach to the same CCR5-targeted ZFNs characterized with the *in vitro* selection approach identified four off-target genomic sites. However, the substantial lack of overlap between off-target sites identified in these two studies (only one site was common to both sets) strongly suggested that neither identified all possible off-target sites. In addition, these results also suggested that a broader range of potential off-target sites might exist beyond the sets identified by these two methods.

Here, we show that *in silico* abstraction of ZFN cleavage profiles generated by the selection method of Liu and colleagues provides an improved approach to screen the human genome for potential ZFN off-target sites. This enhanced strategy identifies both previously described as well as dozens of additional off-target sites for a ZFN pair targeted to *CCR5* gene. We also show that this improved method works effectively for another ZFN pair targeted to the *VEGFA* gene. Our results demonstrate that the potential landscape of off-target mutagenesis effects for ZFNs may be broader than delineated in previous studies.

## MATERIALS AND METHODS

### Plasmids

The plasmids encoding ZFNs targeted to sites in the human *CCR5* (10) and *VEGFA* (11) genes were modified to include heterodimeric EL/KK FokI mutations (12) and were constructed as described in Pattanayak *et al.* (8) (Supplementary Figure S1).

### Processing of *in vitro* selection data

Sequence reads from the *in vitro* cleavage assay reported by Pattanayak were used to generate nucleotide windows comprising the core 9 bp (VEGFA) and 12 bp (CCR5) zinc finger recognition sites as well as the adjacent nucleotides for each ZFN half-site. Sequences shown to cleave efficiently *in vitro* were considered active. The preselection library sequences minus those seen in the active set were considered not efficiently cleaved and labeled as the inactive class. Duplicate entries were removed unless they were identified as independent cleavage events either by experiment or sequence variation in the spacer. Several classifiers including SVMs, decision trees and Naïve Bayes were tested in 10-fold cross-validation analyses using WEKA v3.5.7 (13). Naïve Bayes performed as well or better than the rest of the classifiers and was used exclusively going forward in this study. The test set was built from human genome build HG36 was parsed into similar windows using spacers of 5 and 6 nt separating the two zinc finger half-sites.

### Validation of putative cleavage sites

Individual windows are scored 0 to 1 with windows of lower scores representing sequences that are more likely to be cleaved by the CCR5 ZFNs. K562 cells were treated with catalytically active CCR5-224 ZFNs or a vector-only control, genomic DNA was harvested, and deep sequencing was used to analyze loci of interest as described in Pattanayak *et al*. (8), with the exception that sequencing was carried out for each paired-end library with a 150-cycle MiSeq run (Illumina; Harvard Biopolymers Facility, Boston, MA for CCR5 samples and Dana Farber Cancer Institute for VEGFA samples). Oligonucleotides used to amplify genomic loci of interest are listed in Supplementary Table S10.

### Data processing to identify putative mutagenic NHEJ events

Individual reads were mapped using primer sequences to the individual amplicons and aligned using the Needleman–Wunsch algorithm with affine gap penalties (14). Alignments with <40 bp (minimum combined length of primer) to the reference were excluded, and targets with <1500 reads in either the treated or untreated samples were excluded. Individual alignments were combined to generate a multiple sequence alignment. Identical alignments were counted, condensed and verified to map HG37.57 using BLAT (http://www.kentinformatics.com/). Sequences that mapped preferentially to an alternate target were excluded. Potential NHEJ events required indels of at least 2 nt in length that originated from within the spacer between the ZFN half-sites.

## RESULTS

**Figure 1.**
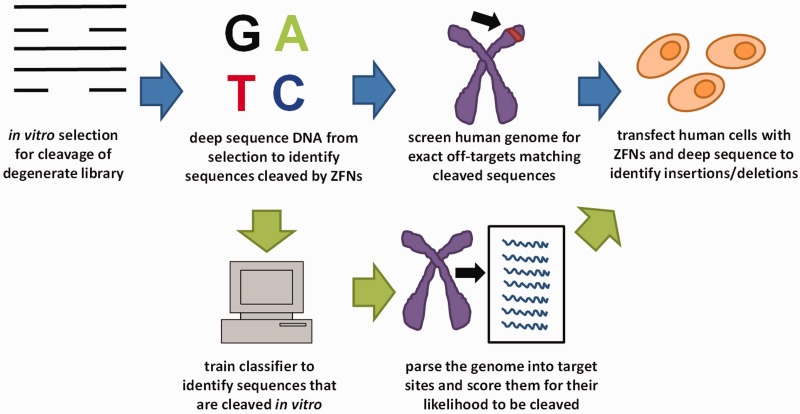
Schematic illustrating the original method by Pattanayak *et al.* (8) (blue arrows) and the enhanced approach that incorporates addition of a classifier-based step (green arrows).

We sought to improve the original strategy of Liu and colleagues by addressing its inability to interrogate cleavage site libraries *in vitro* to a depth sufficient to identify all possible off-target sites present in the human genome. To do this, we added a machine-learning-based step that uses cleavage site preferences from the *in vitro* selection experiments to predict what sequences in the human genome are most likely to be cleaved (Figure 1). We used standard machine-learning techniques to construct Naïve Bayes classifiers that quantify how the nucleotide identity at each position within a DNA site differs between members of a partially degenerate library that were cleaved efficiently *in vitro* and those that were not (‘Materials and Methods’ section). The scores generated by each classifier range from 0 to 1, with lower scores representing a higher probability that any given site will be cleaved (‘Materials and Methods’ section).

We performed an initial test of our approach by developing a classifier based on *in vitro* site selection data previously obtained for ZFNs targeted to a site in the human *CCR5* gene. As shown in Supplementary Table S1, application of this CCR5 ZFN classifier to the human genome resulted in the overwhelming majority of potential target sites having a high classifier score: 11 421 321 184 of 11 421 337 066 potential sites (99.999861%) received a score higher than 0.75. By contrast, only 15 882 sites (0.000139% of all potential sites) had a score lower than 0.75, and only 1123 sites (0.00000983% of all potential sites) had a score below 0.5. Importantly, all 12 *bona fide* off-target sites identified previously by the *in vitro* cleavage site selection, and the IDLV integration methods had scores below 0.75. In addition, 11 of these 12 sites fall within the top 25% of sites with scores below 0.75 (Supplementary Table S2) (8–10).

Having established classifier score cutoffs that enable identification of all previously known off-target sites for the CCR5-targeted ZFNs, we next prospectively tested whether other sites with scores below 0.75 might include additional *bona fide* off-target sites. However, a comprehensive analysis of all sites with scores below 0.75 would require deep sequencing of 15 882 different alleles, an experiment that would be challenging and expensive to perform, given the current cost of next-generation sequencing. Therefore, we instead systematically assessed a smaller sampling of sites by first grouping them based on their position in exonic or non-exonic genomic sequence and then binning sites within each of these groups according to their classifier scores (i.e.—0.0 to 0.1, 0.1 to 0.2, etc.). To achieve high levels of nuclease activity that would facilitate detection of lower frequency off-target events, we used conditions described by Liu and colleagues to overexpress CCR5-targeted ZFNs in K562 cells (‘Materials and Methods’ section). We then used deep sequencing to assess the top 13 scoring sites (if available) within each bin for evidence of NHEJ-mediated indel mutations in the genomic DNA of these cells.

Analysis of 138 sites identified NHEJ-mediated indel mutations not only at the intended CCR5 target site and at a previously known off-target site in the CCR2 gene but also at 21 new off-target sites (Table 1). As expected, the percentage of *bona fide* off-target sites found within each classifier score bin was inversely correlated with the magnitude of the score (i.e.—a greater percentage of actual off-target sites were identified in the lower score bins). For example, 35% (16 of 46) of the screened targets with scores in the first tercile (lowest scores) showed significant evidence of NHEJ-mediated indel mutations compared with 13% (6 of 46) and 2% (1 of 46) of sites with scores in the second and third terciles, respectively (Supplementary Table S3).

**Table 1.** Off-target sites for ZFNs targeted to CCR5 displaying significant evidence of ZFN induced indels grouped by classifier probability score

Probability score	Genomic targets with ZFN indels over total targets screened		Percentage of targets with significant evidence of ZFN-induced indels^a^	Number of targets in human genome scored in this range
	Non-exons	Exons		
0–0.1	0	1/1	100%	1
0.1–0.2	1/4	1/1	40%	5
0.2–0.3	6/12	1/1	54%	60
0.3–0.4	2/12	1/6	17%	241
0.4–0.5	4/12	1/13	20%	816
0.5–0.6	2/12	2/11	17%	2155
0.6–0.7	0/13	0/13	0%	5947
0.7–0.75	0/13	1/13	4%	6657

^a^Significant evidence of ZFN-induced indels as compared with background (controls receiving plasmid with no zinc fingers) was determined using a Fishers exact test and a *P*-value of 0.05.

To test the generalizability of our classifier-based approach, we used it to predict off-target sites for another pair of ZFNs targeted to the human *VEGFA* locus (Supplementary Table S4). Previous work using the *in vitro* cleavage site selection assay had identified a large number of potential off-target sites for this ZFN pair in human cells (Supplementary Table S5) (8). We used this selection data to build a classifier that we used to score every possible site in the human genome (‘Materials and Methods’ section). As we observed with the CCR5 classifier, only a small number (7242) of genomic sites had a classifier score below 0.75, and only 936 sites had a score below 0.5. In addition, all 31 *bona fide* off-target sites identified previously with the *in vitro* selection data all had scores below 0.6, with all but one of these sites having scores below 0.5 (Supplementary Table S6). We assessed 159 potential off-target sites (identified using the same stratified sampling approach we used for the CCR5 ZFNs) for evidence of off-target mutations from genomic DNA of human cells in which the *VEGFA*-targeted nucleases had been expressed. This systematic stratified analysis identified 34 *bona fide* off-target sites, including eight that were previously identified by Pattanayak *et al.* (8) and 26 that were novel (Table 2). We note that that the majority of these novel off-target sites had low classifier scores, again demonstrating the predictive capability of our method (Table 2). Furthermore, several of the sites we predicted to be off-target sites that did not show a statistically significant level of NHEJ mutations in this study had been previously confirmed as off-targets when screened with a greater depth of sequencing reads by Pattanayak *et al.* (8), suggesting that a greater number of the predicted off-target sites might show evidence of mutation with deeper sequence sampling.

**Table 2.** Off-target sites for ZFNs targeted to VEGFA displaying significant evidence of ZFN induce NHEJ grouped by classifier probability score

Probability score	Genomic targets with ZFN indels over total targets screened		Percentage of targets with significant evidence of ZFN-induced indels^a^	Number of targets in human genome scored in this range
	Non-exons	Exons		
0–0.1	3/4	N/A	75%	4
0.1–0.2	6/12	2/2	57%	31
0.2–0.3	5/13	3/6	42%	96
0.3–0.4	4/12	0/13	16%	246
0.4–0.5	2/12	1/12	13%	559
0.5–0.6	2/13	0/13	8%	1187
0.6–0.7	2/12	2/10	9%	2295
0.7–0.75	0/12	3/12	13%	2824

^a^Significant evidence of ZFN-induced indels as compared with background (controls receiving plasmid with no zinc fingers) was determined using a Fishers exact test and a *P*-value of 0.05.

## DISCUSSION

Our results show *in silico* abstraction of *in vitro* cleavage data provides a strategy that more broadly identifies all potential off-target sites of ZFN activity in human cells than previously described methods. Our classifier-based approach not only successfully re-identified all previously known off-target sites for two different ZFNs but also enabled the identification of many additional novel off-target sites, including some that differ from the target sequence by as many as 8 (of 24) or 6 (of 18) bp for the CCR5- or VEGFA-targeted ZFNs, respectively (Tables 3 and 4) (8–10). Because sequences harboring these numbers of mismatches will occur frequently in the human genome, identifying these off-targets would have been previously intractable by simple mismatch counting approaches; indeed, using such strategies would require screening hundreds of thousands of potential sites (Supplementary Tables S7 and S8).

**Table 3.** *In vivo v*alidated off-targets for ZFNs targeted to *CCR5*

Target sequence	Score	Sequence identity to on-target	Observed indel rate	Validating study	Gene	Intron/Exon
GTCATCCTCATCCTGATAAACTGCAAAAG	0.028	24	36.0/40.0%	Perez/Gabriel	CCR5	Exon
			43.5%/47.8%	Pattanayak/Sander		
GTCGTCCTCATCTTAATAAACTGCAAAAA	0.118	22	5.4%/5.8%	Perez/Gabriel	CCR2	Exon
			10.0%/12.9%	Pattanayak/Sander		
TGCTTCCTCACCCCAGGTAAACTGGAACAG	0.133	18	0.25%	Sander		
CTCTCCCTCATCTCAGAGCAACTGTAAAAG	0.204	19	0.39%	Sander		
GCCAGCCTCAGCTTCTTCAACTGGAAAAG	0.209	19	0.07%	Sander		
CTCTTACTCTACATGTTAAACTGAAAAAG	0.215	18	0.06%	Sander	MAP3K7	Intron
CTGGGGCTCAGCACACTCAACTGTAAAAG	0.217	16	0.11%	Sander	DNER	Intron
GCAGTCCTTATCCCAAGTGAACTGAAAAAG	0.219	18	0.05%	Sander	GRP	Intron
CTCTTCCTCAGCATGATTAACTGTAATAG	0.228	18	2.40%	Sander		
GTCCTGCTCAGCAAAAGAAACTGAAAAAG	0.264	20	0.03%	Pattanayak		
GTAGTCCTCCTCCTGCTAAACTGCAATGG	0.270	19	2.13%	Sander		
GACTCCCTCTCCTGGATTAACTGTAAAAG	0.294	17	0.07%	Sander	SKAP2	Exon^a^
GGTGCCCTCACCTTTTTAAACTGTTAAAG	0.306	17	0.60%	Sander	CAMTA1	Intron
GTTATCCTCAGCAAACTAAAACTGGAACAG	0.307	20	0.12%/0.082%	Pattanayak/Sander	WBSCR17	Intron
GGCCTCCTCATCTCTTTAAACTGGAAATG	0.322	20	3.80%	Gabriel		
AAAGTACTCATCCTTTAAGACTGAAAAAG	0.322	17	2.06%	Sander	EREG	Intron
ACATTGCTCATCACAAAGTAACTGTAAAAG	0.342	17	0.81%	Sander		
GTCTTCCTGATGCTACCAAACTGGAAAAG	0.348	20	0.02%	Pattanayak		
TGGTTGCTCATCTCCAAGAAACTGGAAAGG	0.357	17	0.80%	Sander		
CCCCCCCTCATCCCAATTAACTGTAAAAT	0.364	17	0.11%	Sander	VANGL1	Exon
ACACACCTCTTCCTCATAAACTGGAAGAG	0.400	16	4.59%	Sander		
CCCATGCTCTGCCCAGTCAACTGGGAAAG	0.400	16	0.03%	Sander		
TGTGTCCTCTGCATCAGTAAACTGAAACAG	0.401	16	2.50%	Sander	DTD1	Intron
GTGTTGCTTCTCCCCATCAACAGGAAAAG	0.401	16	0.22%	Sander		
GGAGAATGCAGCTTCATAAACTGCAAAAG	0.401	16	0.03%	Sander	FBLIM1	Intron
TGAGACCTCATCTCTTAAAACTGTAATAG	0.405	17	2.40%	Gabriel	KDM2A	Intron
GTCCTCCTCATTCACACAAACTGGAAGGG	0.408	19	0.87%	Sander	IQSEC1	Intron
AGAGGCCTCCTCTCTTTAAACTGTAACAG	0.421	16	0.10%	Gabriel	ZCCHC14	Intron
GGACTCCCTCTCCTGGATTAACTGTAAAAG	0.428	16	0.05%	Sander	SKAP2	Exon^a^
TTGGTCCTCATTATAAATAAACTGAAAGGG	0.500	17	0.02%	Sander		
ATGGACCTCAGCAAAGTAAACTGGAAAAC	0.501	17	0.02%	Sander		
TATTTCCCCATCTCAATAAACTGCAATAG	0.505	18	0.07%	Sander	RAB3IP	Exon
GTTCCCCTCAGCAATGTAAACTGGGAAAC	0.508	17	0.27%	Sander	DOPEY1	Exon
GTTTTCCTCATCAAAGCAAACTGCAAAAT	0.523	21	0.07%	Pattanayak		
GTCATCTTCATCAGCATAAACTGTAAAGT	0.542	20	0.33%	Pattanayak	TACR3	Intron
ATGTTCCTCATCTCCCGAAACTGCAAATG	0.593	20	0.07%	Pattanayak	KCNB2	Intron
GTCAACCTCAACACCTACAGACTGCAAAAG	0.603	21	0.06%	Pattanayak		
GTCATCCTCATCGCCATCAACCGACATGG	0.701	18	0.02%	Sander	MY07B	Exon
GTCATCTTCATCAAAAGGAACTGCAAAAC	0.710	21	0.04%	Pattanayak		

^a^Overlaps with alternate SKAP site.

**Table 4.** *In vivo v*alidated off-targets for ZFNs targeted to *VEGFA*

Target sequence	Score	Sequence identity to on-target	Observed indel rate	Validating study	Gene	Intron/Exon
AGCAGCGTCTTCGAGAGTGAGGA	0.059	18	14.48%/17.66%	Pattanayak/Sander	VEGF-A	Exon
AGCATCGTCTGAAGTGAGTGAGGC	0.076	16	0.66%/1.45%	Pattanayak/Sander		
AGCAAAGTCTGTACTGAGTGAGGG	0.088	15	1.32%	Pattanayak	OPN5	Intron
AGCAACGTCATATTCAGTGAGGA	0.095	16	0.05%/0.19%	Pattanayak/Sander		
AGCAATGTCAAAAAGAGTGAGGC	0.115	15	0.15%/0.24%	Pattanayak/Sander	SIK3	Intron
AGCAGCGTCCTTCCTCAGTGAGAC	0.132	15	0.05%	Sander		
AGCACCGTCCCCCTCAGTGAGGC	0.136	15	0.28%	Pattanayak	PDE9A	Intron
AGCAGCGTATCACATGAGTGAGGG	0.143	16	0.44%/0.99%	Pattanayak/Sander		
AGCAGCGTCTCCCTTGAGTGATGG	0.145	16	0.04%	Pattanayak	PTK2B	Intron
AGCAACTTCATCTTGAGTGAGGG	0.145	15	0.03%	Sander		
AGCACGGTCATGATGAGTGAGGC	0.148	15	0.04%/0.18%	Pattanayak/Sander	PLXNA4	Exon
AGCAGGGTCAGGGCTGAGTGAGGC	0.152	16	0.26%/1.00%	Pattanayak/Sander		
AGCAGCGTCGTGTGGTGTGAGGT	0.155	16	0.40%	Sander	AK8	Intron
AGCATCGTCTTTCTGTGTGAGGC	0.161	15	0.27%	Sander	CTXN3	Exon
AGCAGAGTCAGACTTGAGTGAGGT	0.163	16	0.10%	Pattanayak	LOC550643	Intron
AGCAACGTCCATAGTGTGTGAGAA	0.181	15	0.64%	Pattanayak	GBF1	Intron
GGCAACGTCAACTCAGAGTGAGAA	0.202	15	0.04%	Sander		
AGCAGGGTCACACTAAAGTGAGGC	0.209	15	0.34%	Sander		
AGCAGCGTCTAGGGGGAGGGAGGG	0.209	16	0.56%/0.10%	Pattanayak/Sander	HAUS5	Exon-Intron
AGCAGCGGCCCGCAGAGGGAGGC	0.213	15	2.23%	Sander		
AGCAGTGTCAGCCATGAGGGAGGG	0.216	15	1.81%	Sander	BC04086	Intron
AGCAGCTTCTCCTGGGAGTGAGGG	0.224	16	0.32%	Pattanayak		
AGCAAAGTCCTTGGTAAGTGAGGG	0.225	14	0.79%	Sander	ERMP1	Exon
AGCAGAGTCTCTGAGAGTGAGGC	0.236	16	0.09%	Pattanayak	HEATR8	Intron
AGCATTGTCTCATGTGAGTGAGGT	0.258	15	0.60%	Pattanayak		
AGCACGGTCAGTCTTCAGTGAGGG	0.267	14	0.96%	Sander	EGLN3	Exon
AGCAGCGACGCCTGGGAGTGAGGT	0.268	16	1.11%	Pattanayak		
AGCAGCGGCGGCTGCAGTGAGGC	0.276	15	0.30%	Pattanayak	MTX2	Exon
AGCAGCGGCAGCGAGAGTGATGT	0.285	15	0.06%	Sander	KIF3C	Exon
AGCATTGTCTCCTGGAGTGAGGG	0.294	15	0.05%	Pattanayak		
AGCACAGTCAATCTTCAGTGAGGG	0.301	14	0.05%	Sander	DERA	Intron
AGCTCCGGCAGACATGAGTGAGGG	0.302	14	0.07%	Sander	CDKL3	Intron
AGCATGGTCCCAAGGAGTGAGGG	0.304	15	0.16%/0.21%	Pattanayak/Sander	HRASLS	Intron
GGCAGAGTCAGGGCTGAGTGAGGC	0.305	15	0.03%	Sander	CELF4	Intron
AGCATCGTCTTCTGTGAGTGAGTA	0.314	16	0.06%	Pattanayak	MICAL3	Intron
AGCACCGTGGCTTCGAGTGAGGC	0.339	15	0.03%	Pattanayak		
AGAAACGTCGTGGAGGAGTGAGGG	0.352	15	0.04%	Pattanayak		
AGCAGTGTCAGGCTGGTGTGAGGA	0.361	16	2.10%	Pattanayak		
AGCAGTGTCAGGCTGGTGTGAGGA	0.361	16	2.80%	Pattanayak		
AGCAGCGTGCAGTGACAGTGAGGC	0.400	15	0.04%	Sander	SYT9	Intron
AGCAAGGTCCATCCAGAGAGAGGC	0.402	14	0.22%	Sander	EVL	Intron
AGCAGCGTCTGAAAGAGTGAAAA	0.413	16	0.07%	Pattanayak		
TGCAGCGGCGTAGGGGAGTGAGGA	0.426	16	0.07%	Pattanayak	SARDH	Intron
AGCAGAGTCCAGTGGGTGTGAGGC	0.432	15	0.05%	Sander	SLC22A23	Exon
AGCATAGTCTAGGCCGAGTGAGGC	0.435	15	0.06%	Pattanayak		
AGCAGTGTCAGGCTGGTGTGAGGA	0.461	16	0.34%	Pattanayak		
AGCAGTGTCAGGCTGGTGTGAGGA	0.461	16	0.15%	Pattanayak		
AGCAGTGTCAGGCTGGTGTGAGGA	0.461	16	0.11%	Pattanayak		
AGCAAGGTCCACCAGGTGGGAGGG	0.500	13	0.01%	Sander	CHST11	Intron
AGTAGTGTCTCAGAAGAGGGAGGG	0.501	14	0.61%	Sander	CNBD1	Intron
AGCAGTGTCCTAAGGGGGTGAGGA	0.570	16	0.13%	Pattanayak	SBF2	Intron
AGTAAGGTCACTCATAAGTAAGGT	0.600	12	5.10%	Sander		
TGCAGCGGCGGCGGGAGGGAGGG	0.600	14	0.01%	Sander		
TGCACCGTCAAGAGTCAGTGAGAA	0.605	14	0.04%	Sander	BEND4	Exon
AGCCAGGTCACAGCTGAGAGAGGC	0.613	13	0.02%	Sander	ANGPLT7	Exon
AGCAGCGGCCGCCTGAGGGGAGC	0.701	13	3.11%	Sander	CHAF1A	Exon
AGCAACAGCCCTGGGGGGTGAGGT	0.704	13	0.01%	Sander	FBLN2	Exon
AGCAACTGCGAGCTGGGTGAGGC	0.705	13	0.08%	Sander	PRDZ	Exon

Importantly, because we only assessed a small sampling of the top scoring potential off-target sites in cells, we believe that the full range of potential off-target sites for the two ZFN pairs we examined is likely more expansive than just those identified in this study. This expectation is supported by another experimental screen (data not shown) that identified six additional *bona fide* off-target sites for the CCR5-targeted ZFNs (Supplementary Table S9 and Supplementary Discussion). Collectively, these results clearly demonstrate that ZFN off-target sites may occur at low rates much more widely on a genome-wide scale than suggested by data from previously described reports.

Although our data clearly demonstrate that sites with low classifier scores are highly enriched for *bona fide* off-target sites, our results also show that *bona fide* off-targets are present (albeit at a much lower frequency) among loci with higher classifier scores. This suggests that comprehensive identification of off-target sites will require interrogation of a large number of loci by deep sequencing. We expect that decreases in the price per base and increases in the number of bases that can be sequenced should increase the number of potential sites with low classifier scores that can be examined, thereby enabling the identification of a greater number of *bona fide* off-target sites. However, until such reductions in sequencing costs become reality, an alternative approach might be to look at off-targets with the best scores or to pre-screen off-targets bioinformatically for sites that fall in regions of high priority such as promoters, exons and non-coding RNAs.

We note that the number of off-target sites identified by our approach may be larger or smaller depending on the cell type examined as well as the level and duration of ZFN expression. Not all of the sites with low classifier scores we examined showed evidence of mutagenesis. Potential reasons for this might include DNA methylation of the target site or chromatin status of the gene. These parameters will be cell-type specific and would not be accounted for by *in vitro* selections or *in silico* classifiers. As large-scale efforts such as ENCODE and the NIH Roadmap Epigenomics Mapping Project define these variables in multiple different cell types, it may be possible to use such information to prioritize sites with low classifier scores and thereby to increase the yield of *bona fide* off-target sites identified by deep sequencing. In addition, we expressed ZFNs from a strong constitutive CMV promoter using transiently transfected plasmids and harvested genomic DNA from cells 5 days post-transfection. Lower levels and shorter durations of ZFN expression might be expected to induce fewer off-target mutations, whereas higher levels and longer durations might induce an even greater number of such mutations.

More broadly, the combined strategy of using *in vitro* cleavage site selection data together with machine-learning-based classifiers might also be extended to specificity information from other sources (e.g.–SELEX or bacterial selection) and to define the specificities of nucleases built on other platforms (e.g.—TALENs or CRISPR-Cas RNA-guided nucleases). The use of machine learning to improve the predictive power of data derived from *in vitro* selection experiments could be particularly useful for ZFNs composed of greater numbers of fingers in each monomer and for TALENs. These nucleases target longer sites, making it challenging to adequately sample all potential off-targets even in an *in vitro* system. Continuing to better define off-target effects of targeted nucleases will provide important information to guide refinement of the genome-wide specificities of these reagents. These improvements will be critically important, as these targeted nucleases are more widely applied for both research and therapeutic approaches.

## SUPPLEMENTARY DATA

Supplementary Data are available at NAR Online.

## FUNDING

National Institutes of Health (NIH) Director’s Pioneer Award [DP1 GM105378], [NIH P50 HG005550] and Defense Advanced Research Projects Agency (DARPA) [W911NF-11-2-0056 to J.K.J.]; The Jim and Ann Orr Massachusetts General Hospital (MGH) Research Scholar Award (to J.K.J.); [NIH T32 CA009216 to J.D.S.]; National Science Foundation Graduate Research Fellowship and a Ford Foundation Predoctoral Fellowship (to C.L.R.); National Science Foundation (NSF) ward [DBI-0923827 to D.R.]; [DARPA HR0011-11-2-0003, DARPA N66001-12-C-4207] and the Howard Hughes Medical Institute (to V.P. and D.R.L.); Award number [T32GM007753 to V.P.] from the National Institute of General Medical Sciences. National Human Genome Research Institute Grant [U54 HG004570 to N.S., M.K., B.E.B.]. Funding for open access charge: NIH [DP1 GM105378].

*Conflict of interest statement*: J.K.J. has a financial interest in Transposagen Biopharmaceuticals. J.K.J.’s interests were reviewed and are managed by Massachusetts General Hospital and Partners HealthCare in accordance with their conflict of interest policies.

## Supplementary Material

Supplementary Data
