# Supplementary material for: In silico abstraction of zinc finger nuclease cleavage profiles reveals an expanded landscape of off-target sites
Source: Nucleic Acids Res. 2013 Aug 14;41(19):e181. doi: 10.1093/nar/gkt716 (PMC3799455; doi:10.1093/nar/gkt716)
Supplement: Supplementary Data [file supp_41_19_e181__index.html]

In silico abstraction of zinc finger nuclease cleavage profiles reveals an expanded landscape of off-target sites — In silico abstraction of zinc finger nuclease cleavage profiles reveals an expanded landscape of off-target sites — Supplementary Data 

# *In silico* abstraction of zinc finger nuclease cleavage profiles reveals an expanded landscape of off-target sites

## Supplementary Data

files

**Files in this Data Supplement:**

- Supplementary Data - docx file
- Supplementary Data - docx file
- Supplementary Data - xlsx file
