# Supplementary material for: In silico abstraction of zinc finger nuclease cleavage profiles reveals an expanded landscape of off-target sites
Source: Nucleic Acids Res. 2013 Aug 14;41(19):e181. doi: 10.1093/nar/gkt716 (PMC3799455; doi:10.1093/nar/gkt716)
Supplement: Supplementary Data [file supp_gkt716_nar-00933-h-2013-File003.docx]

**Supplemental Discussion**

*Filtering targets identified by classifier using ChIP*

In an earlier approach to identify ZFN off-target sites we preformed chromatin immunoprecipitation (**ChIP**) to screen for genomic targets. However, ChIP showed little or no evidence of activity at many of the validated off-target sites (including those from this study one). Furthermore, it identified tens of thousands of likely monomer binding sites that showed no evidence of NHEJ when sampled using deep sequencing. This is not necessarily surprising because thousands of high affinity monomeric target sites may exist in the genome, however a monomer is not sufficient to generate a lesion. Alternatively, dimeric ZFN sites that are bound weakly by both monomers may be sufficient to cleave DNA at a low frequency but may not bind stably enough to be detected reliably via ChIP. However, filtering this data using an earlier version of our classifier we identified six loci that showed evidence of ZFN induced indels that also scored well using our classifier. We re-validated these low scoring (most likely to cleave) targets with greater sequence depth again for this study (Supplementary Table 9).
