# Supplementary material for: In silico abstraction of zinc finger nuclease cleavage profiles reveals an expanded landscape of off-target sites
Source: Nucleic Acids Res. 2013 Aug 14;41(19):e181. doi: 10.1093/nar/gkt716 (PMC3799455; doi:10.1093/nar/gkt716)
Supplement: Supplementary Data [file supp_gkt716_nar-00933-h-2013-File004.docx]

Supplemental Figure X. Amino acid sequence ZFNs targeting *CCR5 & VEGFA*

>CCR5 ZFN (+KK)

MDYKDHDGDYKDHDIDYKDDDDKMAPKKKRKVGIHGVPAAMAERPFQCRICMRNFSDRSN

LSRHIRTHTGEKPFACDICGRKFAISSNLNSHTKIHTGSQKPFQCRICMRNFSRSDNLAR

HIRTHTGEKPFACDICGRKFATSGNLTRHTKIHLRGSQLVKSELEEKKSELRHKLKYVPH

EYIELIEIARNSTQDRILEMKVMEFFMKVYGYRGKHLGGSRKPDGAIYTVGSPIDYGVIV

DTKAYSGGYNLPIGQADEMQRYVKENQTRNKHINPNEWWKVYPSSVTEFKFLFVSGHFKG

NYKAQLTRLNHKTNCNGAVLSVEELLIGGEMIKAGTLTLEEVRRKFNNGEINF

>CCR5 ZFN (-EL)

MDYKDHDGDYKDHDIDYKDDDDKMAPKKKRKVGIHGVPAAMAERPFQCRICMRNFSRSDN

LSVHIRTHTGEKPFACDICGRKFAQKINLQVHTKIHTGEKPFQCRICMRNFSRSDVLSEH

IRTHTGEKPFACDICGRKFAQRNHRTTHTKIHLRGSQLVKSELEEKKSELRHKLKYVPHE

YIELIEIARNSTQDRILEMKVMEFFMKVYGYRGKHLGGSRKPDGAIYTVGSPIDYGVIVD

TKAYSGGYNLPIGQADEMERYVEENQTRNKHLNPNEWWKVYPSSVTEFKFLFVSGHFKGN

YKAQLTRLNHITNCNGAVLSVEELLIGGEMIKAGTLTLEEVRRKFNNGEINF

>VEGFA ZFN (+KK)

MDYKDHDGDYKDHDIDYKDDDDKMAPKKKRKVGIHGVPSRPGERPFQCRICMRNFSRQDR

LDRHTRTHTGEKPFQCRICMRNFSQKEHLAGHLRTHTGEKPFQCRICMRNFSRRDNLNRH

LKTHLRGSQLVKSELEEKKSELRHKLKYVPHEYIELIEIARNSTQDRILEMKVMEFFMKV

YGYRGKHLGGSRKPDGAIYTVGSPIDYGVIVDTKAYSGGYNLPIGQADEMQRYVKENQTR

NKHINPNEWWKVYPSSVTEFKFLFVSGHFKGNYKAQLTRLNHKTNCNGAVLSVEELLIGG

EMIKAGTLTLEEVRRKFNNGEINF

>VEGFA ZFN (-EL)

MDYKDHDGDYKDHDIDYKDDDDKMAPKKKRKVGIHGVPSRPGERPFQCRICMRNFSTGQI

LDRHTRTHTGEKPFQCRICMRNFSVAHSLKRHLRTHTGEKPFQCRICMRNFSDPSNLRRH

LKTHLRGSQLVKSELEEKKSELRHKLKYVPHEYIELIEIARNSTQDRILEMKVMEFFMKV

YGYRGKHLGGSRKPDGAIYTVGSPIDYGVIVDTKAYSGGYNLPIGQADEMERYVEENQTR

NKHLNPNEWWKVYPSSVTEFKFLFVSGHFKGNYKAQLTRLNHITNCNGAVLSVEELLIGG

EMIKAGTLTLEEVRRKFNNGEINF
